# Prioritization of Vaccines for Introduction in the National Immunization Program in the Republic of Korea

**DOI:** 10.3390/vaccines12080886

**Published:** 2024-08-04

**Authors:** Won Suk Choi, Yeonhee Sung, Jimin Kim, Hyeri Seok, Young J. Choe, Chelim Cheong, Jahyun Cho, Dong Woo Lee, Jee Yeon Shin, Su-Yeon Yu

**Affiliations:** 1 Division of Infectious Diseases, Department of Internal Medicine, Korea University Ansan Hospital, Korea University College of Medicine, Ansan 15355, Republic of Korea; cmcws@korea.ac.kr (W.S.C.); hyeri.seok@gmail.com (H.S.); 2 Research Support Team, Korea University Research & Business Foundation, Seoul 02841, Republic of Korea; dough117w@gmail.com; 3 Division for Healthcare Technology Assessment Research, National Evidence-Based Healthcare Collaborating Agency, Seoul 04933, Republic of Korea; jimin@neca.re.kr; 4 Department of Pediatrics, Korea University Anam Hospital, Korea University College of Medicine, Seoul 02841, Republic of Korea; choey@korea.ac.kr; 5 Department of Pharmacy, College of Pharmacy, Kangwon National University, Chuncheon 24341, Republic of Korea; chelimc@gmail.com; 6 Graduate School of Public Health, Seoul National University, Seoul 08826, Republic of Korea; jahyun04@snu.ac.kr; 7 Division of Immunization, Bureau of Healthcare Safety and Immunization, Korea Disease Control and Prevention Agency, Osong 28159, Republic of Korea; wiliamdongwoolee@korea.kr (D.W.L.); shinjeeyeon@korea.kr (J.Y.S.)

**Keywords:** national immunization program, prioritization, framework, criteria, Delphi survey

## Abstract

This study presents a framework for determining the prioritization of vaccine introduction in the National Immunization Program (NIP) of the Republic of Korea, with a focus on case examples assessed in 2021 and 2023. We describe the predefined criteria for evaluating the prioritization of vaccines in the NIP and the established process in the Republic of Korea. These criteria included disease characteristics, vaccine characteristics, rationality and efficiency of resource allocation, and the acceptance of immunization. The process of prioritizing NIP introduction involved several sequential steps: a demand survey, evidence collection, preliminary evaluation, priority evaluation, and decision making. In 2021 and 2023, 14 and 25 committee members participated in evaluating the prioritization of vaccines in the NIP, respectively. Overall, 13 and 19 NIP vaccine candidates were included in the 2021 and 2023 evaluations, respectively. Through the Delphi survey and consensus processes, the priority order was determined: vaccination against Rotavirus infection was the top priority in 2021, while Influenza 4v (for chronic disease patients) took precedence in 2023. This study demonstrates an evidence-based decision-making process within the healthcare field. The outlined approach may provide valuable guidance for policymakers in other countries seeking to prioritize the inclusion of new vaccines in their NIP.

## 1. Introduction

In recent times, with the spread of the coronavirus disease 2019 pandemic worldwide, countries have been earnestly dedicated to the development of vaccines. Subsequently, they have experienced a series of developmental and regulatory processes aimed at supporting a larger population receiving vaccinations. There are more than 25 vaccines currently available to prevent diseases, protect health throughout the lifespan, and prevent and mitigate outbreaks [1]. There are ongoing efforts to develop vaccines with improved clinical efficacy and safety, even for diseases where vaccines have already been developed. In this context, determining which vaccines to include in the National Immunization Program (NIP) poses a new challenge from a government health policy perspective. The inclusion of a vaccine in the NIP leads to a large number of individuals receiving vaccinations annually. This has significant implications regarding public health, as considerable budgets are repeatedly allocated for vaccine administration and adverse event management.

Burchett et al. conducted a systematic review with 85 articles to summarize the decision-making systems, principles, and case studies involved in introducing new vaccines into NIPs [2]. They derived nine factors that influence a country’s decision-making process: the importance of health issues, vaccine characteristics, feasibility, acceptability, accessibility and equity, financial and economic considerations, the impact of immunization, alternative treatment situations, and decision-making procedures [2]. The World Health Organization also suggests three factors that should be considered in determining the introduction of vaccines into NIPs: disease characteristics, vaccine characteristics, and programmatic capacities [3]. Disease characteristics encompass public health priorities, disease burden, and the effectiveness of disease prevention and control strategies. Vaccine characteristics include efficacy and safety, cost, cost-effectiveness, and the availability of a stable vaccine supply. Finally, programmatic capacities indicate the need for successful vaccine introductions and a sustainable healthcare system to support long-term operations. Therefore, when making decisions regarding the introduction of new vaccines, it is crucial to consider not only the introduction of the vaccine but also the potential impact on the overall healthcare system.

In the Republic of Korea (hereafter Korea), the early system of the NIP was established with the enactment of the Communicable Diseases Prevention Act in 1954, designating vaccination for seven infectious diseases as routine immunization. The current framework for the NIP has been in place since the widespread adoption of standard vaccination guidelines in 1997. As of 2023, preventive vaccinations are conducted for a total of 14 infectious diseases [4]. In Choi et al.’s study, conducted in Korea, a Delphi survey was conducted among members of the Korea Expert Committee on Immunization Practices (KECIP), which acts as the Advisory Committee on Immunization Practices in the USA [5]. This survey focused on the prioritization of five vaccine candidates (Haemophilus influenzae type b, Hepatitis A, Pneumococcal, Rotavirus, and Human papillomavirus vaccine) for inclusion in the NIP [5]. Key considerations at that time included disease characteristics (incidence rate, mortality rate, severity, disease burden, and epidemic potential) and vaccine characteristics (effectiveness, cost, and international status). The Pneumococcal, Haemophilus influenzae type b, and Hepatitis A vaccines were evaluated as having a high priority for introduction [5]. Subsequently, the authors highlighted the need for systematic literature reviews and evidence-based evaluations in the decision-making process for vaccine introduction in Korea [6]. Furthermore, a recent version of this study presented the results of expert consensus on the evaluation system and principles for the introduction of vaccines in the NIP [7].

This study aims to present the principles and evaluation system for the introduction of vaccines in Korea’s NIP, with a focus on case examples that assessed the prioritization of vaccine introduction in 2021 and 2023. Moreover, it aims to discuss potential areas of improvement for the future.

## 2. Methods

In 2021 and 2023, prioritization for vaccine introduction was conducted according to predefined principles for the evaluation of the NIP and the established process in Korea. This study was approved by the Institutional Review Board of Korea University (IRB No. 2023AS0066).

### 2.1. Principles for the Evaluation of the National Immunization Program Introduction

Kim et al. (2019) suggested four principles for the introduction of new vaccines in the NIP [7]: disease characteristics, vaccine characteristics, rationality and efficiency of resource allocation, and acceptance of immunization. Our study operationalized each principle by specifying their detailed review items and data sources, as shown in Table 1.

### 2.2. Priority-Setting Process

**Figure 1:**
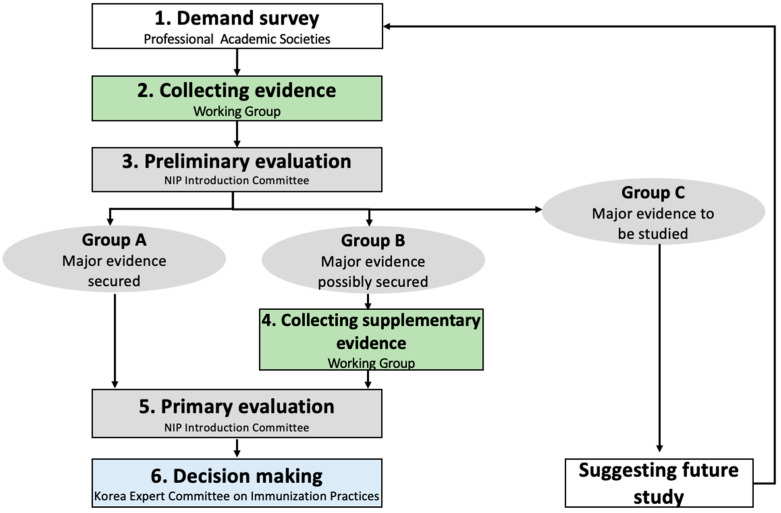
Schematic flow of the prioritization of the introduction of vaccines to the National Immunization Program in the Republic of Korea.

The process of determining the priority for introduction in the NIP involved several sequential steps. Initially, a demand survey was conducted to identify candidate vaccines with the participation of professional academic societies, including the Korean Society of Internal Medicine, the Korean Pediatric Society, and the Korean Society of Infectious Diseases, among others. In the demand survey, professionals did not consider which vaccines were included because the environments related to the need for vaccines are constantly changing. Following this, the working group, composed of specialists in evidence-based methodology, systematically gathered and analyzed evidence regarding the NIP vaccine candidates, adhering to established criteria for the evaluation of NIP introduction: disease characteristics, vaccine characteristics, rationality and efficiency of resource allocation, and acceptance of immunization. Following this, the NIP Introduction Committee, consisting of relevant experts, conducted a preliminary evaluation, categorizing vaccines into groups based on the availability of supporting evidence: acquisition of all evidence related to criteria shown in Table 1 (Group A); potential short-term acquisition of major evidence (Group B); and need for long-term acquisition of major evidence, such as cost-effectiveness analysis using Korean data (Group C). Additional evidence for Group B was sought before the prioritization process. Vaccines requiring long-term evidence gathering (Group C) were then excluded from the priority evaluation. Among the criteria and items, disease prevalence, vaccine approval, cost-effectiveness, budget, and acceptability should be derived from Korean data. For Group A and Group B, the NIP Introduction Committee conducted a primary evaluation to determine the priority for NIP inclusion. Thereafter, the Korea Expert Committee on Immunization Practices (KECIP) meticulously reviewed the entire process and outcomes of the priority-setting process, ultimately making the final decision. The specific methods for each step are detailed according to the sequence illustrated in Figure 1.

### 2.3. Primary Evaluation: Delphi Method and Data Analysis

**Figure 2:**
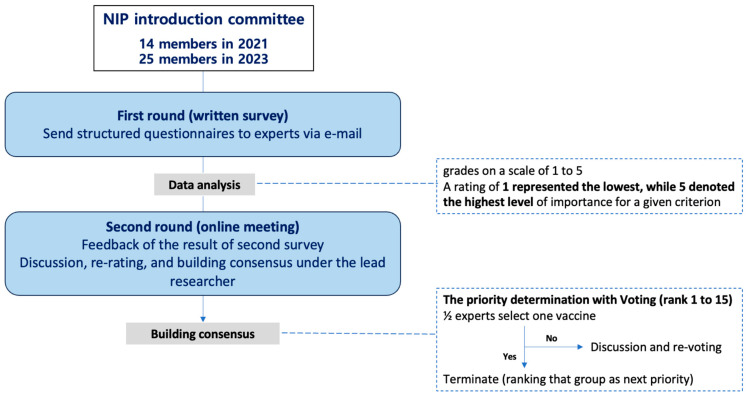
Primary evaluation process for prioritizing National Immunization Program vaccines (modified Delphi consensus method).

In the primary evaluation aimed at prioritizing vaccines for potential inclusion in the NIP, we employed a modified Delphi technique facilitated by the NIP Introduction Committee (Figure 2). This committee comprised experts in various fields, including public health, infectious disease, epidemiology, health economics, immunology, health statistics, and clinical medicine, with 14 and 25 members in 2021 and 2023, respectively. The evaluation process consisted of two rounds of expert consultation, utilizing electronic questionnaires for the first round and real-time video meetings for the second round. Both rounds were anonymously self-administered.

During the first round, committee members were tasked with assessing each vaccine candidate by assigning grades on a scale of one to five, indicating the importance of factors within the provided frameworks (Table S1). A rating of one represented the lowest, while five denoted the highest level of importance for a given criterion. We conducted a comprehensive analysis, calculating frequencies, means, medians, minimum values, and maximum values for the importance scores of indicators at each level, considering each vaccine in the evaluation.

In the second round, the aggregated results from the initial survey were shared as statistics with all members. Members were then required to prioritize vaccines for inclusion in the NIP through real-time online surveys conducted multiple times until all vaccine candidates were determined. For instance, in the initial survey, the vaccine voted as the highest priority was considered to have higher prioritization and was excluded from the subsequent surveys.

## 3. Results 

### 3.1. Participants

In 2021, during the preliminary evaluation, 11 out of 14 committee members (78%) completed the questionnaire, while all 14 members actively participated in the primary evaluation for the prioritization of vaccines in the NIP. Among the members, three (21%) were public health officers, three (21%) were experts in epidemiology or public health, and eight (57%) were professionals in clinical fields related to vaccine-preventable diseases. Some experts also served as members of the Korea Expert Committee on Immunization Practices (KECIP) subcommittees. 

In 2023, during the preliminary evaluation, 22 out of 25 committee members (88%) completed the questionnaire, while all 25 members actively participated in the primary evaluation for the prioritization of vaccines in the NIP. Among the members, one (4%) was a public health officer, four (16%) were experts in epidemiology or public health, and twenty (80%) were professionals in clinical fields related to vaccine-preventable diseases. Some experts also served as members of KECIP subcommittees. 

### 3.2. NIP Vaccine Candidates and Preliminary Evaluation

To select the vaccines for review, a demand survey was conducted among experts from various medical societies, including 16 regional medical societies, the Korean Association of General Practice, 22 specialty societies, the Korean Medical Association, and 26 specialty medical societies in 2021 and 2023. 13 and 19 NIP vaccine candidates were included in the 2021 and 2023 evaluations, respectively. 

The preliminary evaluation is a crucial step in determining the presence of key evidence required for the subsequent processes of primary priority evaluation for the introduction of vaccines in the NIP. The main purpose is to classify vaccines for which priority evaluation cannot be conducted based on the current evidence. The NIP candidates and the preliminary evaluation results are described in Table 2. 

The NIP Introduction Committee conducted a preliminary evaluation, categorizing vaccines into groups based on the availability of supporting evidence. In the preliminary evaluation, we classified the eight vaccines (2021) and fifteen vaccines (2023) as having secured key evidence (Group A) or having the potential for short-term key evidence acquisition (Group B; Table S2).

### 3.3. Prioritization of NIP Vaccines

Table 3 presents the average scores assigned to each NIP vaccine candidate and target population based on the disease-related, vaccine-related, resource allocation, and acceptability frameworks. Concerning the disease factor, considering prevalence and mortality, Pneumococcal (4.55) was identified as the most significant health problem in 2021, while influenza (4.43) ranked the highest in 2023. Regarding the vaccine factor, the Rotavirus vaccine (4.64) was rated the highest for having significant efficacy in preventing disease in 2021, whereas the Recombinant Zoster vaccine (4.46) held the top position in 2023. Regarding resource allocation and acceptability factors, the unmet needs of the Rotavirus vaccine were deemed the highest (4.73), and its inclusion in the NIP was considered the second most cost-effective (4.18) in 2021. However, in 2023, the influenza vaccination for patients with chronic diseases took precedence (4.23).

As a result of the Delphi survey and consensus process, the following priority order was determined: in 2021, Rotavirus > Influenza (chronic disease patients) > Influenza (50–64 years) > Hepatitis A (13–18 years) > Pneumococcal (PCV13) > Hepatitis A (19–39 years) > Influenza (13–18 years) > Varicella; in 2023, Influenza 4v (chronic disease patients) > Pneumococcal (PCV13) > HPV 9v (girls) > Zoster (live) > adjuvant high dose Influenza (65 years and more) > HPV 9v (boys and girls) > Influenza 4v (50–64 years old) > Hepatitis A (19–49 years) > Influenza 4v (13–18 years) > Varicella > Hepatitis A (13–18 years old) > Tdap/Td (boosting) > Zoster (live, recombinant) > HPV 4v (boy) > Zoster (recombinant).

### 3.4. Decision Making

The KECIP comprehensively reviewed the entire process and outcomes of the priority-setting process, ultimately making the final decision on when and which vaccine would be added to the NIP, considering factors such as resource availability and public health impact. In the 2021 priority setting, the highest priority was the Rotavirus vaccine. Thereafter, the KECIP decided that the Rotavirus vaccine would be introduced into the NIP in 2023, providing the target population with the benefits of free preventive vaccination. However, the KECIP has not yet made a decision regarding the 2023 priority setting.

## 4. Discussion 

In this study, we presented two experiences of determining the introduction of vaccines on priority in the NIP in Korea through expert consensus processes based on a scientific framework. In 2021, the highest priority was given to the introduction of the Rotavirus vaccine into the NIP, followed by the second-highest priority for the introduction of the quadrivalent influenza vaccine for individuals aged 19–64 years with chronic conditions. As a result, the Rotavirus vaccine was introduced into the NIP in 2023, providing the population with the benefits of free preventive vaccination. In the 2023 priority evaluation, the quadrivalent influenza vaccine for individuals aged 19–64 years with chronic conditions again held the highest priority, indicating a decision-making process similar to that in the previous assessment. The prioritization for vaccine introduction into the NIP proposed in this study was derived through an expert consensus process based on the evidence collected thus far. Therefore, the priority evaluation of NIP vaccine candidates may change in the future, considering the generation of new evidence or administrative feasibility of the program, such as vaccine supply availability and budgetary considerations, which could lead to a modification in the sequence of vaccine introductions.

Several previous studies have investigated national decision-making processes regarding the prioritization of vaccine introductions [2,8,9,10,11,12,13]. According to a recent systematic review, commonly reported criteria for decision-making were the burden of disease, vaccine efficacy/effectiveness and safety, impact on health and non-health outcomes, economic evaluation, and cost-effectiveness [9]. Programmatic and acceptability aspects were not as frequently considered. However, our criteria included an assessment of the acceptability of vaccination, which was based on the status of optional vaccination and overseas NIP, social value, equity, and feasibility. The data sources are derived from expert opinions and government policy, as well as quantitative data. This indicates that although the decision making for NIP prioritization in Korea strives for an objective process based on scientific evidence achieved through the agreement of NIP committee members, the Korean government is likely to influence prioritization decisions regarding vaccine introduction from the perspective of acceptability. Additionally, previous studies have emphasized the importance of economic evaluations in prioritization processes [2]. Similarly, in our two experiences of prioritizing NIP vaccine introductions, candidate vaccines lacking economic evaluations were excluded from the main evaluation process. 

In this study, we did not address which specific principle or criterion among the vaccine introduction principles is the most important. The importance of each principle can vary depending on the perspectives of stakeholders (such as the government, healthcare professionals, and patients) and the societal context (including the economic, political, and healthcare environment) [9,13,14]. Reaching a common agreement on which principle is the most important is a challenging task. Therefore, previous studies on decision making regarding vaccine introduction have provided a general direction for evaluating the introduction of new vaccines nationally but found it difficult to clarify explicit criteria. Moreover, different countries have varying issues regarding infectious diseases, vaccines, and policy and environmental factors, and even within a country, the prioritization considerations may differ depending on the vaccine. Therefore, the focus of evaluation can vary, and the establishment of vaccine policies requires a systematic and comprehensive approach, preferably based on evidence [15]. Consequently, the decision-making process for the introduction of new vaccines or the expansion of target populations and vaccine types should ensure transparency, be based on scientific evidence, and consider the characteristics of national public health.

As the available data sources for analysis and societal demands for vaccines are likely to continue evolving, it is necessary to continuously revise and enhance the process of introducing vaccines into the NIP while considering their applicability in the Korean context. Vaccines introduced in the NIP are used by many people over a long period of time and require substantial resources annually. Therefore, the fundamental principle of evaluating the priority of vaccine introduction based primarily on scientific evidence should not be compromised. In Korea, there are currently numerous vaccines recommended by professional societies but not yet included in the NIP, as well as many new vaccines targeting a large population and awaiting market release. Consequently, there is a high likelihood of strong demand for the expansion of the NIP in the future. Therefore, evaluations for the introduction of vaccines into the NIP should be conducted at least every one to three years to accommodate these potential changes.

Regarding coronavirus disease 2019 (COVID-19) vaccination, in Korea, COVID-19 vaccines have been provided to citizens for free via a special program, not through the NIP so far. This is because, while the NIP is based on a routine and regular vaccination plan, the environment surrounding COVID-19 has been changing, such as transmission, prevalence, mortality, vulnerable groups, vaccine efficacy, etc. [16,17,18]. However, a recent study supported employing annual preventative measures against SARS-CoV-2, such as administering seasonal booster vaccines in a similar timeframe in a similar timeframe as those in place for influenza [19]. Therefore, the Korean government and specialists also need to consider including the COVID-19 vaccine in the NIP.

The NIP is primarily managed by government bodies such as the Korea Disease Control and Prevention Agency (KDCA) in Korea. Government intervention ensures standardized procedures and widespread accessibility. In contrast, in some low- and middle-income countries, non-governmental organizations (NGOs) play a crucial role in vaccine distribution, often filling gaps left by insufficient public health infrastructure [20]. The collaboration between governments and NGOs can enhance the reach and efficiency of vaccination programs, leveraging the strengths of both sectors. Additionally, the introduction of vaccines into a National Immunization Program (NIP) is influenced by cultural and societal contexts, which vary significantly across different regions [2,3,21,22]. In Korea, high literacy rates and a strong public health infrastructure support the efficient dissemination and acceptance of vaccines. However, in other parts of the world, cultural and religious beliefs can significantly impact vaccination acceptance [2,22]. For instance, certain communities may have reservations about vaccines due to religious doctrines or historical mistrust of medical interventions. Policymakers must tailor vaccination strategies to address these unique cultural contexts to ensure successful immunization programs [3,21]. 

Vaccine hesitancy remains a significant barrier to achieving high vaccination coverage [23,24]. Strategies to counteract this include public education campaigns, transparent communication about vaccine safety and efficacy, and engagement with community leaders to endorse vaccination. Digital transformation plays a pivotal role in the introduction of vaccines. E-health platforms facilitate the collection of real-time data, monitor vaccine distribution, and enhance communication between healthcare providers and the public [25,26,27]. In Korea, digital tools have streamlined the appointment scheduling process, reminder systems, and data management, contributing to the high efficiency of the NIP. The use of these technologies can be especially beneficial in tracking immunization coverage and identifying areas with low vaccination rates.

This study had several limitations typical of qualitative studies, including Delphi surveys. First, the inherent regression to the mean is a weakness of this survey method. The data may be influenced by the potential unwillingness of experts to express certain opinions to their peers despite the survey being conducted anonymously. Second, the current evidence collected and used by the committee members to prioritize NIP vaccines is likely to be biased. However, we tried to reduce this bias through a systematic and predefined literature search and presented representative nationwide statistics. Third, despite efforts to ensure representation by including experts from various fields, the inclinations of the NIP introduction evaluation committee members are likely to influence the outcomes of determining priorities, significantly. Therefore, it is crucial to select committee members who possess expertise and ensure their independence. Furthermore, the extent to which the members understand the evaluation process can also impact priority decisions. Therefore, after selecting the introduction evaluation committee members, it would be beneficial to provide them with some level of guidance and simulation to facilitate a smooth evaluation.

Nevertheless, we believe that the findings of this survey merit consideration for informing the integration of optional vaccines into the NIP in Korea, as well as potentially in other countries facing comparable circumstances. In this study, we modified and supplemented the vaccine introduction principles and criteria proposed in a previous study [7]. We also suggest the use of reliable and representative data sources and methodology to support the evidence for each vaccine. The process we have introduced may assist policymakers in other countries by guiding them on how to prioritize a new vaccine for inclusion in NIPs. Future research should focus on cross-country comparisons to understand varied approaches to vaccine prioritization and introduction. Comparative studies in diverse contexts can provide valuable insights, helping to identify best practices and inform global health policies.

## Figures and Tables

**Table 1:** Criteria for the evaluation of National Immunization Program introduction.

Category	Criteria	Item	Source
Disease	The disease should be an important health problem, and vaccination should be an important and final disease control measure.	Prevalence Mortality Disability weight Transmission rate Pandemic possibility Importance of vaccine as a disease control tool	Literature review Data analysis Expert opinion
Vaccine	The safety of the vaccine has been proven, with the vaccine having significant efficacy in preventing disease.	Approval Efficacy and safety Patient safety report Clinical practice guideline	Literature review Data analysis Web search
Resource allocation	Vaccine introduction should be rational and the allocation of resources should be efficient.	Cost-effectiveness Budget	Literature review Data analysis
Acceptability	The public should be able to accept the vaccination.	Optional vaccination International National Immunization Program Other considerations (social value, equity, feasibility, etc.)	Data analysis Web search Expert opinion Government policy

**Table 2:** National Immunization Program vaccine candidates and preliminary evaluation.

Vaccine		Target Population	2021		2023	
			Candidate	Group	Candidate	Group
Influenza	Adjuvant/ high dose/ recombinant	≥65 years old	-	-	Yes	A
	4-valent	50–64 years old	Yes	A	Yes	B
		With chronic disease (2021)/ 19–64 years old with chronic disease (2023)	Yes	B	Yes	A
		13–18 years old	Yes	A	Yes	A
TDaP/Td	-	≥20 years old	Yes	C	Yes	A
TDaP	-	Pregnant women	Yes	C	-	-
Hepatitis A	-	19–39 years old (2021)/ 19–49 years old (2023)	Yes	B	Yes	A
	-	13–18 years old	Yes	B	Yes	A
Pneumococcal	PCV13	≥65 years old	Yes	A	Yes	A
	PCV15	≥65 years old	-	-	Yes	C
		19–64 years old with underlying disease	-	-	Yes	C
	PCV20	≥65 years old	-	-	Yes	C
		19–64 years old with underlying disease	-	-	Yes	C
Herpes zoster	ZVL or RZV	≥65 years old	Yes	A	-	-
	ZVL	≥70 years old	-	-	Yes	B
	RZV		-	-	Yes	B
	ZVL or RZV		-	-	Yes	B
Rotavirus	-	2, 4, (6) months old	Yes	A	-	-
Varicella 2nd dose	-	4–6 years old	Yes	C	Yes	A
Human papilloma virus	4-valent	11–12-year-old boys	Yes	C	Yes	B
	9-valent	11–12-year-old girls	Yes	C	Yes	B
	9-valent	11–12 years old	-	-	Yes	B

Note: Group A, evidence secured; Group B, potential short-term acquisition of major evidence; Group C, need for long-term acquisition of major evidence. Abbreviations: PCV, Pneumococcal conjugate vaccine; RZV, Recombinant Zoster Vaccine; ZVL, Zoster Vaccine Live; 4-valent, quadrivalent; 9-valent, nonavalent.

**Table 3:** Prioritization of vaccines to be included in the National Immunization Program according to evaluation based on the Delphi survey (2021 and 2023).

Vaccine		Target Population	2021						2023					
			Disease	Vaccine		Resource Allocation	Acceptability	Rank	Disease	Vaccine		Resource Allocation	Acceptability	Rank
				Efficacy	Safety					Efficacy	Safety			
Influenza	Adjuvant/ high dose/ recombinant	≥65 years old	-	-	-	-	-	-	4.43 (0.65)	3.93 (0.62)	4.07 (0.73)	3.93 (0.62)	4.43 (0.51)	5
	4-valent	50–64 years old	3.91 (1.22)	4.00 (0.45)	4.55 (0.52)	4.00 (0.89)	4.09 (0.54)	3	4.43 (0.65)	4.07 (0.47)	4.43 (0.51)	4.07 (0.73)	4.21 (0.70)	7
		With chronic disease (2021)/19–64 years old with chronic disease (2023)	4.09 (1.14)	4.09 (0.54)	4.27 (0.65)	4.27 (0.90)	4.18 (0.75)	2	4.15 (0.90)	4.15 (0.55)	4.38 (0.51)	4.23 (0.44)	4.31 (0.48)	1
		13–18 years	3.45 (1.13)	3.73 (0.65)	4.45 (0.52)	3.55 (0.93)	3.73 (0.65)	7	4.07 (1.07)	3.79 (0.89)	4.43 (0.51)	3.71 (1.14)	4.00 (0.88)	9
TDaP/Td	-	≥ 20 years old	-	-	-	-	-	-	3.36 (0.93)	4.00 (0.88)	4.07 (0.62)	3.93 (0.73)	3.86 (0.86)	12
Hepatitis A	-	19–39 years old (2021)/19–49 years old (2023)	3.91 (0.83)	4.45 (0.69)	4.45 (0.69)	3.18 (0.60)	3.64 (0.81)	6	4.00 (0.82)	4.08 (0.49)	4.23 (0.44)	3.31 (0.75)	3.62 (0.65)	8
	-	13–18 years old	3.45 (0.69)	4.27 (0.90)	4.36 (0.81)	3.18 (0.87)	3.55 (0.82)	4	3.86 (0.77)	4.21 (0.58)	4.36 (0.50)	3.43 (0.85)	4.00 (0.78)	11
Pneumococcal	PCV13	≥65 years old	4.55 (0.52)	3.45 (0.52)	4.09 (0.54)	3.55 (0.82)	3.55 (0.93)	5	4.31 (0.63)	4.15 (0.38)	4.31 (0.48)	4.15 (0.55)	4.31 (0.48)	2
Herpes zoster	ZVL or RZV	≥65 years old	2.73 (1.35)	3.82 (0.75)	4.00 (0.77)	3.36 (1.12)	4.18 (0.60)	8	-	-	-	-	-	-
	ZVL	≥70 years old	-	-	-	-	-	-	3.36 (0.84)	4.00 (0.78)	4.07 (0.73)	3.36 (1.08)	4.21 (0.43)	4
	RZV		-	-	-	-	-	-	3.23 (0.73)	4.46 (0.66)	4.08 (0.64)	3.46 (0.97)	4.15 (0.38)	15
	ZVL or RZV		-	-	-	-	-	-	3.38 (0.77)	4.00 (0.82)	3.85 (0.90)	3.15 (1.14)	4.08 (0.76)	13
Rotavirus	-	2, 4, (6) months old	4.00 (0.77)	4.64 (0.50)	4.36 (0.50)	4.18 (0.40)	4.73 (0.47)	1	-	-	-	-	-	-
Varicella 2nd dose	-	4–6 years old	-	-	-	-	-	-	3.86 (0.66)	4.00 (0.39)	4.00 (0.39)	3.21 (0.97)	4.07 (0.83)	10
Human papilloma virus	4-valent	11–12-year-old boys	-	-	-	-	-	-	3.71 (0.99)	3.86 (0.66)	4.07 (0.62)	3.21 (1.05)	3.86 (0.66)	14
	9-valent	11–12-year-old girls	-	-	-	-	-	-	4.23 (0.93)	4.38 (0.51)	4.31 (0.48)	3.62 (1.04)	4.23 (0.6)	3
	9-valent	11–12 years old	-	-	-	-	-	-	3.77 (1.17)	4.08 (0.95)	4.08 (0.76)	3.31 (1.03)	4.00 (0.91)	6

Abbreviations: PCV, Pneumococcal conjugate vaccine; RZV, Recombinant Zoster Vaccine; ZVL, Zoster Vaccine Live; 4-valent, quadrivalent; 9-valent, nonvalent.

## Data Availability

The original contributions presented in the study are included in the article/Supplementary Materials, further inquiries can be directed to the corresponding author.

## Supplementary Materials

The following supporting information can be downloaded at: https://www.mdpi.com/article/10.3390/vaccines12080886/s1, Table S1. (A). National Immunization Program Vaccine Candidates and Preliminary Evidence Evaluation: Disease, Vaccine, and Cost-effectiveness. (B). National Immunization Program Vaccine Candidates and Preliminary Evidence Evaluation: Acceptability; Table S2: Result of the Preliminary Evidence Evaluation.
