# Correction: Choi et al. Prioritization of Vaccines for Introduction in the National Immunization Program in the Republic of Korea. *Vaccines* 2024, *12,* 886

**DOI:** 10.3390/vaccines13090912

**Published:** 2025-08-28

**Authors:** Won Suk Choi, Yeonhee Sung, Jimin Kim, Hyeri Seok, Young J. Choe, Chelim Cheong, Jahyun Cho, Dong Woo Lee, Jee Yeon Shin, Su-Yeon Yu

**Affiliations:** 1 Division of Infectious Diseases, Department of Internal Medicine, Korea University Ansan Hospital, Korea University College of Medicine, Ansan 15355, Republic of Korea; cmcws@korea.ac.kr (W.S.C.); hyeri.seok@gmail.com (H.S.); 2 Research Support Team, Korea University Research & Business Foundation, Seoul 02841, Republic of Korea; dough117w@gmail.com; 3 Division for Healthcare Technology Assessment Research, National Evidence-Based Healthcare Collaborating Agency, Seoul 04933, Republic of Korea; jimin@neca.re.kr; 4 Department of Pediatrics, Korea University Anam Hospital, Korea University College of Medicine, Seoul 02841, Republic of Korea; choey@korea.ac.kr; 5 Department of Pharmacy, College of Pharmacy, Kangwon National University, Chuncheon 24341, Republic of Korea; chelimc@gmail.com; 6 Graduate School of Public Health, Seoul National University, Seoul 08826, Republic of Korea; jahyun04@snu.ac.kr; 7 Division of Immunization, Bureau of Healthcare Safety and Immunization, Korea Disease Control and Prevention Agency, Osong 28159, Republic of Korea; wiliamdongwoolee@korea.kr (D.W.L.); shinjeeyeon@korea.kr (J.Y.S.)

The authors would like to make the following corrections to this published paper [1].

The authors regret the omission of an appropriate citation, in Table 1 and Figure 1, of the work by Kim, H. S. et al., cited as reference [7] in the original publication. The legends for Table 1 and Figure 1 have now been updated by adding the citation. The correct legend appears below:

Table 1. Criteria for the evaluation of National Immunization Program introduction. The criteria and items were proposed by Kim et al. (2020) [7].

Figure 1. Schematic flow of the prioritization of the introduction of vaccines to the National Immunization Program in the Republic of Korea. The figure is based on the process proposed by Kim et al. (2020) [7].

There were errors in the original publication regarding (1) the publication year and the title of reference [7] cited in Methods, Section 2.1, and (2) the omission of reference [7] citation in Methods, Section 2.2. The corrected reference [7] should be as follows: 

Kim, H.S.; Park, J.H.; Eun, B.W.; Choi, W.S.; Korea Disease Control and Prevention Agency. Development of an Evaluation Framework for New Vaccine Introduction. 2020. Available online: https://library.nih.go.kr/ncmiklib/archive/rom/reportView.do (accessed on 1 May 2024).

The correct Section 2.1 paragraph 1, first sentence appears below:

Kim et al. (2020) suggested four principles for the introduction of new vaccines in the NIP [7]: disease characteristics, vaccine characteristics, rationality and efficiency of resource allocation, and acceptance of immunization.

The following sentence should be inserted as the second sentence in Methods, Section 2.2. Priority-Setting Process:

“This multi-step process was based on the evaluation framework originally developed by Kim et al. (2020) [7]”.

The authors state that these changes do not affect the scientific conclusions of the article. This correction was approved by the Academic Editor. The original publication has also been updated.
