# Supplementary material for: Prioritization of Vaccines for Introduction in the National Immunization Program in the Republic of Korea
Source: Vaccines (Basel). 2024 Aug 4;12(8):886. doi: 10.3390/vaccines12080886 (PMC11359589; doi:10.3390/vaccines12080886)
Supplement: Supplementary file 1 [file vaccines-12-00886-s001.zip › vaccines-3091042-supplementary/Table S1.pdf]

Table S1 (A). National Immunization Program Vaccine Candidates and Preliminary Evidence Evaluation: Disease, Vaccine, and Cost-effectiveness

No	Purpose	Criteria	Disease					Vaccine			Cost-effectiveness
			Prevalence/ Mortality	Disability weight	Transmission rate	Pandemic possibility	Importance of vaccine for disease control	Efficacy/ Safety		Patient safety report	
		Evidence resource	National statistics*	Previous study	Previous study	-	-	Approval data	RCT, RWD	KID, KDCA	Previous study in Korea
		Research methods	Data analysis	Literature review		Expert opinion		Literature review	Rapid SR	Data analysis	Rapid SR
1		HZ live: ≥ 70 years old								O	
2	New introduction	HZ recombinant: ≥ 70 years old	O	O	NA	O	O	O	O	X	O
3		HZ live or recombinant: ≥ 70 years old								△	
4	Expansion of target population	HPV4: 12-year-old boys									
5	Addition of vaccine type	HPV9: 12-year-old girls	O	O	O	O	O	O	O	O	O
6	Expansion of target population and addition of vaccine type	HPV9: 12-year-old boys and girls									
7	Addition of vaccine type	Influenza (adjuvant/high dose/recombinant): ≥ 65 years old						O		X	
8		Influenza (quadrivalent): 50–64 years old	O	O	O	O	O		O		O
9	Expansion of target population	Influenza (quadrivalent): 19–64 years old with chronic disease						X		O	
10		Influenza (quadrivalent): 13–18 years old									
11	Expansion of target population	Tdap/Td: ≥ 20 years old	O	O	T: NA<br>D/aP: O	O	O	O	X	O	O
12		PCV13: ≥ 65 years old						O	O	O	O
13	Addition of vaccine type	PCV15: ≥ 65 years old									
14		PCV20: ≥ 65 years old						O			
15		PCV15 > PPSV23: 19–64 years old with underlying disease	O	O	O	O	O		X	X	X
16	New introduction	PCV 23: 19–64 years old with underlying disease						X			
17	2nd dose introduction	Varicella 2nd dose: 4–6 years old	O	O	O	O	O	O	O	O	O
18	Catch-up vaccination	Hepatitis A: 19–49 years old	O	O	O	O	O	O	X	O	O
19		Hepatitis A: 13–18 years old									

Notes. O, Full evidence; △, Specific evidence only; X, Absence of evidence. *National Statistics included Infectious Disease Statistics in KDCA, National Health Insurance Claim Data, and National mortality statistic. Abbreviations: HZ, herpes zoster; HPV, human papillomavirus; KDCA, Korea Disease Control and Prevention Agency; KIDS, Korea Institute of Drug Safety & Risk Management; NIP, national immunization program; NA, not available; PCV, Pneumococcal Conjugate Vaccine; PPSV, Pneumococcal polysaccharide vaccine; RCT, randomized controlled trial; RWD, real-world data; SR, systematic review; Tdap, Tetanus-Diphtheria-Pertussis; Td tetanus-diphtheria.

Table S1 (B). National Immunization Program Vaccine Candidates and Preliminary Evidence Evaluation: Acceptability

No	Purpose	Criteria	Acceptability		
			Optional vaccine use rate	International NIP	Other considerations
		Evidence resource	Supply data, NIP data	Official website	Academic opinion or policy
		Research methods	Data analysis	Web search	Survey and search
1	New introduction	HZ live: ≥ 70 years old	O	O	- (Demand Survey) KAIM 1st<br>- National policy: New HZ vaccine for seniors<br>- ‘23 National audit opinion
2		HZ recombinant: ≥ 70 years old			
3		HZ live or recombinant: ≥ 70 years old			
4	Expansion of target population	HPV4: 12-year-old boys	O	O	- (Demand Survey) KUA 1st, KSPiD 3rd, KPS 4th, KPA† 1st<br>- National policy<br>- Presidential election pledge<br>- ‘22, 23 National audit opinion
5	Addition of vaccine type	HPV9: 12-year-old girls			
6	Expansion of target population and addition of vaccine type	HPV9: 12-year-old boys and girls			
7	Addition of vaccine type	Influenza (adjuvant/high dose/recombinant): ≥ 65 years old	X	O	- (Demand Survey) KAIM 1st (KSiD)<br>- (International) Recommendation for immunization<br>- ‘22,’23 National audit opinion
8	Expansion of target population	Influenza (quadrivalent): 50–64 years old	O		-
9		Influenza (quadrivalent): 19–64 years old with chronic disease			- ‘22 National audit opinion
10		Influenza (quadrivalent): 13–18 years old			- (Demand Survey) KSPiD 5th, KPS 3rd
11	Expansion of target population	Tdap/Td: ≥ 20 years old	O	O	- (Demand Survey) KAIM 2nd (KSiD)<br>- (Demand Survey) KPA‡† 3rd
12	Addition of vaccine type	PCV13: ≥ 65 years old	O	O	-
13		PCV15: ≥ 65 years old	X	O	
14		PCV20: ≥ 65 years old			
15	New introduction	PCV15 > PPSV23: 19–64 years old with underlying disease			- (Demand Survey) KAIM 3rd (KSiD)
16		PCV 23: 19–64 years old with underlying disease			- (Demand Survey) KAIM 3rd (KSiD)
17	2nd dose introduction	Varicella 2nd dose: 4–6 years old	O	O	- (Demand Survey) KSPiD 2nd, KPS 5th
18	Catch-up vaccination	Hepatitis A: 19–49 years old	O	O	- (Demand Survey) KAIM 4th (KSiD)
19		Hepatitis A: 13–18 years old			- (Demand Survey) KSPiD 1st, KPS 2nd

Notes. O, Full evidence; △, Specific evidence only; X, Absence of evidence. Abbreviations: HZ, herpes zoster; HPV, human papillomavirus; KAIM, Korean Association of Internal Medicine; KPA†, Korean Pediatric Association; KPA‡, Korean Physician’s Association; KPS, Korean Pediatric Society; KSiD, Korean Society of Infectious Diseases; KSPiD, Korean Society of Pediatric Infectious Diseases; NIP, national immunization program; PCV, Pneumococcal Conjugate Vaccine; PPSV, Pneumococcal polysaccharide vaccine; RCT, randomized controlled trial; RWD, real-world data; SR, systematic review; Tdap, Tetanus-Diphtheria-Pertussis; Td tetanus-diphtheria.
