# Supplementary material for: Prioritization of Vaccines for Introduction in the National Immunization Program in the Republic of Korea
Source: Vaccines (Basel). 2024 Aug 4;12(8):886. doi: 10.3390/vaccines12080886 (PMC11359589; doi:10.3390/vaccines12080886)
Supplement: Supplementary file 1 [file vaccines-12-00886-s001.zip › vaccines-3091042-supplementary/Table S2.pdf]

Table S2. Result of the Preliminary Evidence Evaluation

No	Purpose	Candidate		A	B	C	No response
1	New introduction	HZ live: ≥ 70 years old		15	6	1	
2		HZ recombinant: ≥ 70 years old		13	6	2	1
3		HZ live or recombinant: ≥ 70 years old		11	7	3	1
4	Expansion of target population	HPV4: 12-year-old boys		11	7	4	
5	Addition of vaccine type	HPV9: 12-year-old girls		13	5	3	1
6	Expansion of target population and addition of vaccine type	HPV9: 12-year-old boys and girls		10	7	4	1
7	Addition of vaccine type	Influenza (adjuvant/high dose/recombinant)	≥ 65 years old	11	8	3	
8	Expansion of target population	Influenza (quadrivalent)	50-64 years old	14	6	1	1
9			19-64 years old with chronic disease	18	3	0	1
10			13-18 years old	11	7	3	1
11	Expansion of target population	Tdap/Td: ≥ 20 years old		16	3	3	
12	Addition of vaccine type	PCV13: ≥ 65 years old		16	3	2	1
13		PCV15: ≥ 65 years old		2	5	14	1
14		PCV20: ≥ 65 years old		2	4	15	1
15	New introduction	PCV15 > PPSV23: 19–64 years old with underlying disease		2	4	15	1
16		PCV 23: 19–64 years old with underlying disease		1	4	16	1

17	2nd dose introduction	Varicella 2nd dose: 4–6 years old	17	3	2	
18	Catch-up vaccination	Hepatitis A: 19–49 years old	11	7	3	1
19		Hepatitis A: 13–18 years old	11	7	4	

Abbreviation. HZ, herpes zoster; HPV, human papillomavirus; PCV, Pneumococcal Conjugate Vaccine; PPSV, Pneumococcal polysaccharide vaccine; Tdap, Tetanus-Diphtheria-Pertussis; Td tetanus-diphtheria.
